# Supplementary material for: Effect of Mg Addition and PMMA Coating on the Biodegradation Behaviour of Extruded Zn Material
Source: Materials (Basel). 2023 Jan 11;16(2):707. doi: 10.3390/ma16020707 (PMC9863199; doi:10.3390/ma16020707)
Supplement: Supplementary file 1 [file materials-16-00707-s001.zip › materials-2130120-supplementary.pdf]

Supplementary Materials:

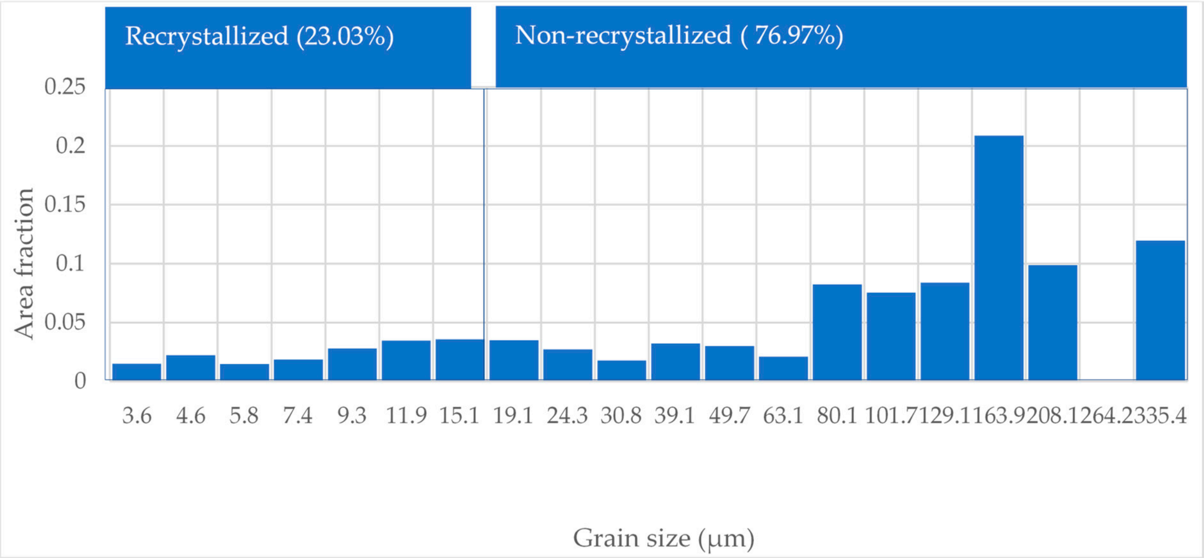

(a)

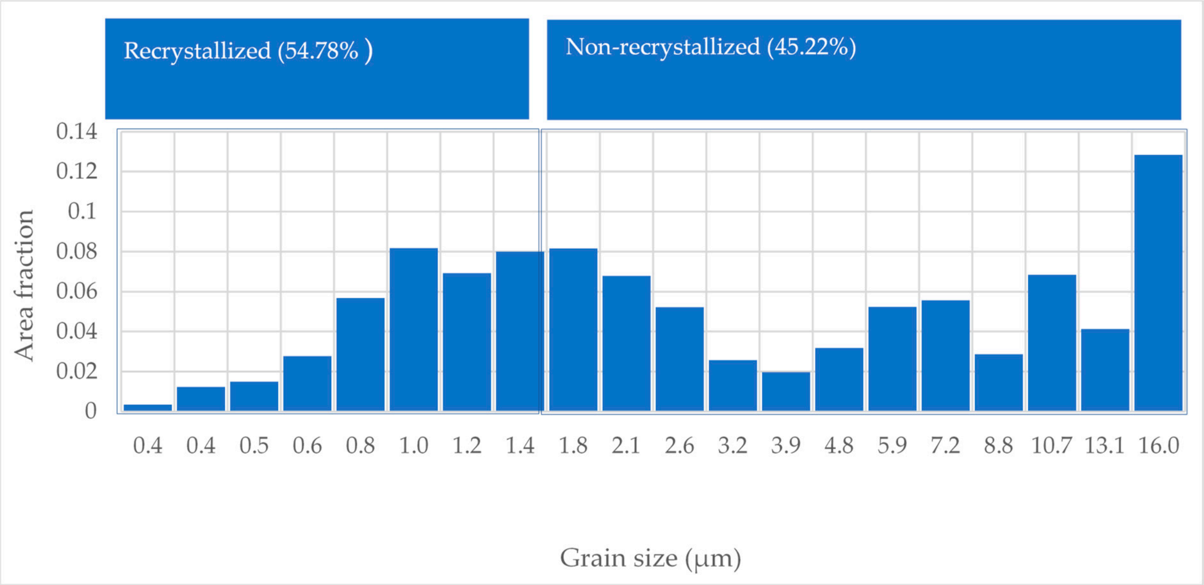

(b)

Figure S1: Grain size distribution related to the corresponding area fraction of: (a) pure Zn; (b) Zn-1.89Mg.

Table S1: Elemental analysis of the eutectic phase of points 1 and 2 in Figure 2.

**EDX analysis**			
		**Zn**	**Mg**
**1**	wt.%	98.96	1.04
**dark phase**	at.%	97.24	2.76
**2**	wt.%	97.57	2.43
**light phase**	at.%	93.72	6.28

Table S2: Elemental analysis of the points 1 - 6 in Figure 8.

EDX analysis											
**Point**		**Zn**	**Mg**	**Br**	**Ca**	**Cl**	**K**	**P**	**Na**	**O**	**C**
**1**	wt.%	66.53	-	-	-	10.09	-	-	-	23.38	-
	at.%	36.96	-	-	-	10.14	-	-	-	53	-
**2**	wt.%	79.36	-	-	-	-	-	-	-	20.64	-
	at.%	48.47	-	-	-	-	-	-	-	51.53	-
**3**	wt.%	47.45	-	-	4.77	2.27	-	7.49	0.01	38.01	-
	at.%	20.58	-	-	3.38	1.82	-	6.86	0.01	67.36	-
**4**	wt.%	83.22	-	-	0.88	-	-	0.26	-	10.05	-
	at.%	59.49	-	-	1.03	-	-	4.04	-	29.36	-
**5**	wt.%	-	-	-	8.51	0.36	-	4.59	-	32.01	54.48
	at.%	-	-	-	3.07	0.15	-	2.14	-	29.00	66.92
**6**	wt.%	-	-	0.31	1.2	0.91	0.49	1.07	-	18.59	77.43
	at.%	-	-	0.05	0.39	0.33	0.16	0.45	-	15.06	83.56

Table S3: Elemental analysis of the points 1 - 6 in Figure 9.

EDX analysis											
**Point**		**Zn**	**Mg**	**Br**	**Ca**	**Cl**	**K**	**P**	**Na**	**O**	**C**
**1**	wt.%	66.69	-	-	4.77	2.27	-	7.49	0.01	18	0.84
	at.%	38.63	-	-	3.32	2.43	-	9.16	0.02	42.61	2.65
**2**	wt.%	70.01	-	-	9.22	0.89	-	8.28	0.01	10.37	1.22
	at.%	45.69	-	-	9.82	1.07	-	11.41	0.02	27.66	4.33
**3**	wt.%	43.5	2.54	-	1.55	4.57	-	6.24	0.01	34.53	7.06
	at.%	20.18	3.17	-	1.17	3.91	-	6.11	0.01	65.45	15.12
**4**	wt.%	4.33	0.28	-	42.74	2.73	1.04	26.91	-	21.97	-
	at.%	1.9	0.33	-	30.56	2.21	0.76	24.9	-	39.35	-
**5**	wt.%	9.01	0.39	1.54	3.14	2.32	0.35	2.47	0.01	42.96	37.81
	at.%	2.21	0.26	0.31	1.26	1.05	0.14	1.28	0.01	43.04	50.46
**6**	wt.%	26.68	0.96	0.52	7.48	2.76	0.39	6.21	0.01	37.07	17.92
	at.%	8.61	0.83	0.14	3.94	1.64	0.21	4.23	0.01	48.90	31.49

Table S4: Elemental analysis of the points 1 – 3 in Figure 10.

EDX analysis											
**Point**		**Zn**	**Mg**	**Br**	**Ca**	**Cl**	**K**	**P**	**Na**	**O**	**C**
**1**	wt.%	83.22	3.17	-	0.88	-	-	2.68	-	10.05	-
	at.%	59.49	6.09	-	1.03	-	-	4.04	-	29.36	-

**2**	wt.%	47.53	1.22	-	0.76	1.01	0.22	1.23	0.02	48.01	-
	at.%	18.78	1.3	-	0.49	0.74	0.15	1.03	0.02	77.51	-
**x3**	wt.%	18.73	0.56	0.53	2.61	0.43	0.67	3.61	-	14.19	58.67
	at.%	4.55	0.37	0.11	1.04	0.19	0.27	1.85	-	14.08	77.55

Topography of corroded surfaces

The topography of the surfaces after removing the corrosion products was illustrated in Figure S2. A pure Zn solution immersed for 240 or 720 hours appears to have very similar topography. The surfaces are composed of peaks and valleys that don’t exceed 4 μm above or below the arithmetic mean line, with no significant pores. Compared to pure Zn, the alloy Zn-1.89Mg exhibits a rougher topography. After 240 h of immersion, scattered pores could be found on the surface of the alloy, the largest of which is 168.60 μm width and 4.58 μm depth below the arithmetic mean. These pores covered more surface area of the alloy and became deeper after immersion for 720 h, some reached 156.29 μm width and ~10.00 μm depth below the arithmetic mean. For the coated alloy after immersion, the roughness does not exceed 1 μm above the arithmetic mean and 3 below it. Removing the swollen parts of the coated samples that were formed after 720 h of immersion left large but shallow holes. Some of these holes reached 213.39 μm in width and 1.95 μm depth.

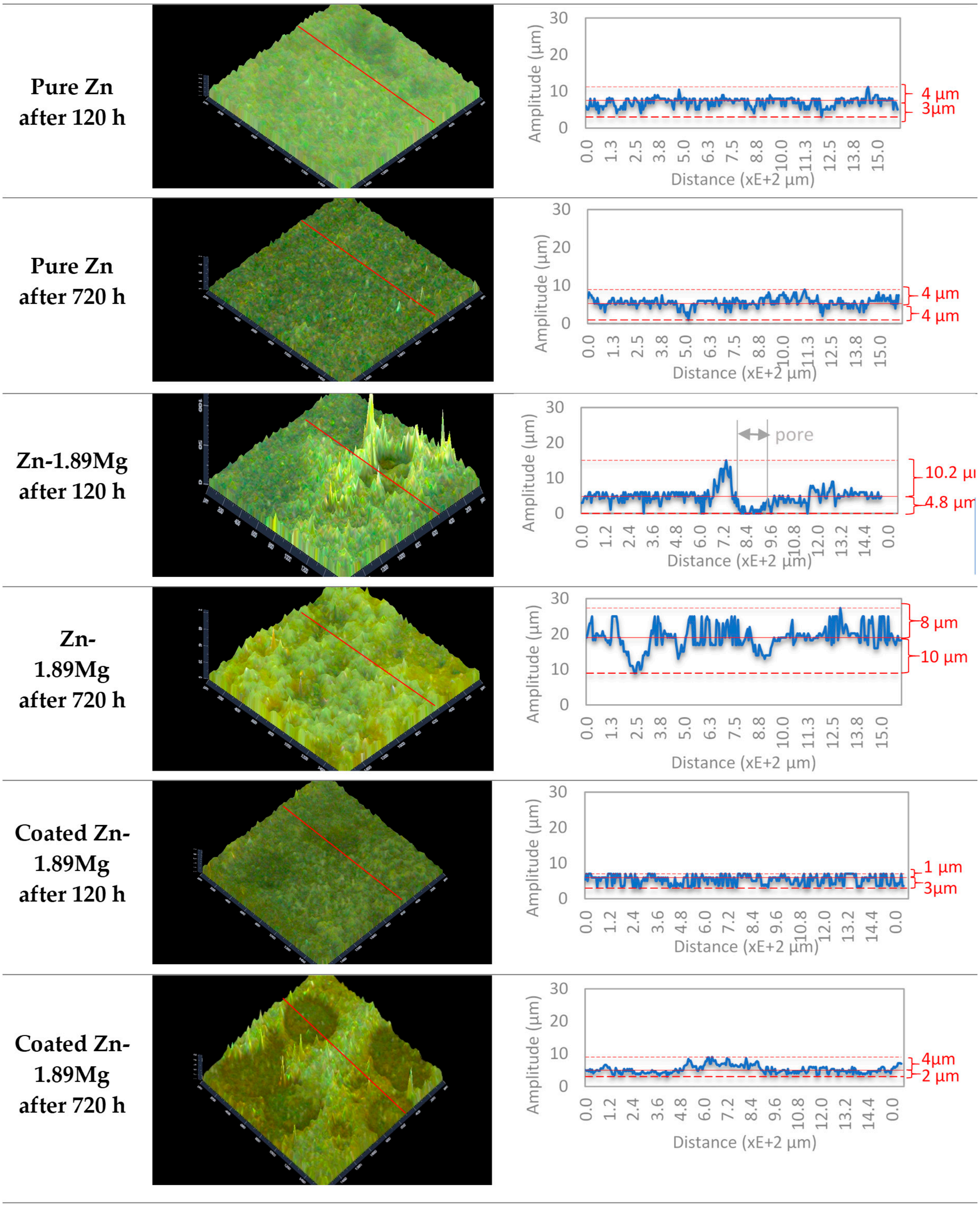

Figure S2. Topography of corroded surfaces after removing the corrosion products.
